# Prenatal Sonographic Features of Noonan Syndrome: Case Series and Literature Review

**DOI:** 10.3390/jcm13195735

**Published:** 2024-09-26

**Authors:** Chayada Tangshewinsirikul, Duangrurdee Wattanasirichaigoon, Thipwimol Tim-Aroon, Patama Promsonthi, Poomiporn Katanyuwong, Sanpon Diawtipsukon, Nareenun Chansriniyom, Theera Tongsong

**Affiliations:** 1 Department of Obstetrics and Gynecology, Faculty of Medicine Ramathibodi Hospital, Mahidol University, Bangkok 10400, Thailand; 2 Department of Pediatrics, Faculty of Medicine Ramathibodi Hospital, Mahidol University, Bangkok 10400, Thailand; 3 Department of Obstetrics and Gynecology, Faculty of Medicine, Chiang Mai University, Chiang Mai 50200, Thailand

**Keywords:** Noonan syndrome, prenatal diagnosis, sonographic features, ultrasound

## Abstract

Noonan syndro me is a rare autosomal dominant congenital abnormality associated with a gene defect located on the short arm of chromosome 12. It is characterized by dysmorphic facies, webbed neck, short stature, lymphatic obstruction, cardiac anomalies, and intellectual disability. Prenatal diagnosis of Noonan syndrome is rare because there are no pathognomonic sonographic signs. Studies on the prenatal sonographic features of Noonan syndrome have been reported in very limited numbers. This case series of severe fetal Noonan syndrome, together with a literature review, was conducted to establish prenatal sonographic features highly suggestive of Noonan syndrome to facilitate early detection by clinicians. This study reveals that Noonan syndrome has a relatively specific pattern, which facilitates prenatal molecular genetic diagnosis. Increased nuchal translucency (NT) in the late first trimester and fluid collection in the early second trimester could be warning signs for follow-up, prompting further investigation to detect late-onset features and leading to molecular genetic confirmation. Most structural abnormalities appear in the second trimester, with progressive changes noted throughout gestation. This review better characterizes the sonographic features of fetal Noonan syndrome based on a larger sample size, illustrating a wider spectrum of prenatal phenotypes, including lymphatic drainage disorders, cardiac abnormalities, polyhydramnios, and absent ductus venosus.

## 1. Introduction

Noonan syndrome (NS; OMIM 163950) is a multisystem disorder, involving RAS-related pathology (RASopathy) [1,2]. Noonan syndrome was first described by Jacqueline Noonan in 1963 [3]. The prevalence of Noonan syndrome is estimated to be 1 in 1000–2500 in the general population [4]. It is inherited in an autosomal dominant manner but two-thirds of patients are the first affected person in their family due to a de novo pathogenic variant [5,6]. Approximately half of patients have a pathogenic variant in protein tyrosine phosphatase, nonreceptor type 11 (PTPN11), with clustering of variants in specific codons. The PTPN11 gene encodes the protein Src homology region 2 domain-containing phosphatase 2 (SHP2), a dual-specificity phosphatase [5], while the remainder of cases are usually associated with a pathogenic variant in one of many other genes encoding a protein of the Ras-mitogen-activated protein kinase (Ras-MAPK) pathway. Noonan syndrome is clinically and genetically heterogeneous. The classical features include typical craniofacial appearances, cardiac anomalies (mainly pulmonary stenosis and hypertrophic cardiomyopathy), restricted growth or short stature, a broad or webbed neck, and variable degree of neurodevelopmental delay [1,7,8]. However, postnatal phenotypic expression ranges from severe to nearly asymptomatic [9].

Prenatal diagnosis of Noonan syndrome has been made in a very limited number of cases. Typically, sonographic features raise a possibility of the disease, prompting either prenatal molecular diagnosis or postnatal confirmation using a clinical scoring system or genetic tests. Before 2001, diagnosis of Noonan syndrome was exclusively clinical, typically based on a comprehensive scoring system [10], whereas molecular diagnosis is currently available and has been increasingly used to confirm the diagnosis. Unfortunately, most clinical findings suggestive of Noonan syndrome do not include prenatal features, though they are often documented prenatally. Nevertheless, several fetal sonographic features can provide diagnostic clues for Noonan syndrome, especially unexplained increased NT, which is associated with Noonan syndrome in 10.5% of cases [11]. Several authors have suggested that Noonan syndrome should be listed in the differential diagnosis of the euploid fetuses with thickened NT, especially in cases of concomitant cardiac defects, polyhydramnios, or fluid collection in any body space [12,13,14,15,16,17,18]. Nevertheless, although many case reports and series have been published, the frequencies of fetal phenotypes were heterogeneous and varied from study to study. For examples, Baldassarre et al. [19] and Menashe et al. [20] showed that prenatal ultrasonography had a low detection rate of fetal anomalies associated with Noonan syndrome, whereas Lamouroux et al. [21] and Hakami et al. [22] showed a high prevalence of structural abnormalities. The discrepancy was likely caused by a small sample size in each study. To date, studies on prenatal sonographic features of Noonan syndrome have been reported in limited numbers and are needed to accumulate cases for comprehensive analysis to specify the sonographic pattern that could help clinicians to recruit cases for proper genetic testing. Accordingly, we reported this cases series of fetal Noonan syndrome together with systematic review to establish prenatal sonographic features highly suggestive of Noonan syndrome.

## 2. Methods

This descriptive study consists of two parts: a case series and literature review. The study was ethically approved by the Institutional Review Boards, Faculty of Medicine Ramathibodi Hospital, Mahidol University, Thailand (Research ID: MURA2024/368).

### 2.1. Case Series

Cases with a diagnosis of fetal Noonan syndrome collected at Ramathibodi Hospital and Maharaj Nakorn Chiang Mai Hospital between 2010 and 2024 were recruited. The inclusion criteria are as follows: (1) a newborn who was prenatally suspected or confirmed to have Noonan syndrome, either based on a molecular genetic test of RASopathy or typical clinical features assessed and confirmed by pediatric geneticists; (2) available prenatal detailed ultrasound (images and video clips) by MFM specialists. All sonographic features were reviewed by the authors. The sonographic features of each pregnancies were categorized and analyzed.

### 2.2. Literature Review

The standard databases, including PubMed, Scopus, and Web of Science, were accessed to perform an electronic search to retrieve original publications of prenatal diagnosis of Noonan syndrome, including both case reports and case series, covering reports from 1980 to 2022. The key words used for the title search were (Noonan syndrome) AND (fetal or prenatal). The consecutive retrieved reports were manually screened based on the abstracts or full texts to recruit only the reports with available detailed sonographic findings and postnatal confirmation of Noonan syndrome. From each report, demographic data, various sonographic findings, and obstetric/neonatal outcomes were validated and extracted by the authors for analysis. Abnormal ultrasound findings thar were not mentioned in the report were interpreted as negative findings. The total cases extracted from the literature were combined with our case series mentioned above. Reports that had no details of prenatal ultrasound findings were excluded. For data preparation, all sonographic findings in all reports were extracted and entered in the data file as separated variables. The sonographic findings that were examined and validated in the first trimester were thickened nuchal translucency (TNT) and cystic hygroma, while those in the second and third trimesters included all abnormal findings. In the analysis, abnormal ultrasound findings were mainly categorized into two groups: the first trimester group and second/third trimester group. The frequencies of each abnormal sonographic finding were analyzed and presented as percentages.

## 3. Results

### 3.1. Case Series (Summarized and Presented in Table 1)

**Figure 1:**
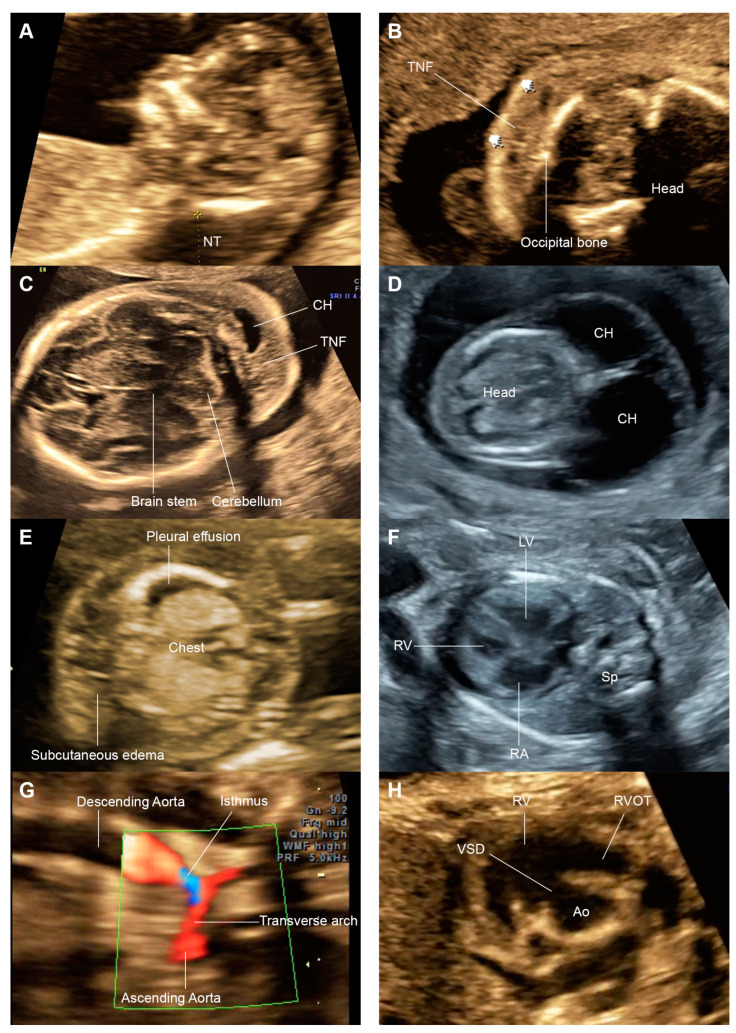
(**A**) Thickened nuchal translucency (NT); (**B**) thickened nuchal fold (TNF); (**C**) TNF and cystic hygroma (CH); (**D**) cystic hygroma (CH); (**E**) hydrops fetalis; (**F**) hypertrophic cardiomyopathy (LV: left ventricle, RA: right atrium; RV: right ventricle, Sp: spine); (**G**) coarctation of the aorta on sagittal scan of the aortic arch; (**H**) ventricular septal defect (VSD) on short-axis view (Ao: aorta; RV: right ventricle; RVOT: right ventricular outflow).

During the study period, a total of 13 cases, including 10 cases at Ramathibodi Hospital and 3 cases at Maharaj Nakorn Chiang Mai Hospital, diagnosed with Noonan syndrome with prenatal sonographic features were available for analysis. Fetal outcome revealed early neonatal death in five cases (38.5%). The mean ± SD maternal age was 34.1 ± 5.1 years. The diagnosis of Noonan syndrome was based on clinical assessment by pediatric geneticists in five cases and molecular genetic testing in eight cases, including PTPN11 (4) and other (4), as presented in Table 1 and Supplementary Table S1. The examples of sonographic features of this series are presented in Figure 1.

Of nine cases undergoing first trimester ultrasound for NT measurements, six (66.7%) had increased NT. Sonographic features of the second trimester are detailed as follows. Cardiac abnormalities were most commonly identified, in 12 out of 13 cases (92.3%), followed by thickened nuchal fold (10/13; 76.9%), the presence of fluid collection in at least one body space (9/13; 69.2%), and cystic hygroma (9/13; 69.2%). Other common findings included polyhydramnios (5; 38.5%) and hydrops fetalis (6; 46.2%). Of note, among cases with cardiac anomalies, hypertrophic cardiomyopathy (HCM) (as an example shown in Figure 1) or ventricular hypertrophy (VH) was identified in up to seven out of 13 cases, followed by pulmonic stenosis (PS) (4) and ventricular septal defect (VSD) (4). Other conditions, which are rarely encountered in general population but were demonstrated in this series, included absent ductus venosus (5; 38.5%), pyelectasis, and single umbilical artery (SUA).

This series indicated that severe Noonan syndrome in Thai fetuses exhibits a specific pattern of sonographic features that are were consistent with those observed in most western studies.

**Table 1:** Summary of the prenatal sonographic features of fetuses with Noonan syndrome in this series.

No.*	GA	TNT	CH	TNF	Cardiac Defect	Hydrops Fetalis	Pleural Effusion	Subcutaneous Edema	Ascites	Pericardial Effusion	Polyhydramnios	Short Femur	LGA	SUA	Pyelectasis	Absent DV	Others	Diagnosis	Neonatal Notes
1	13,20	y	y	y	HCM, ostium primum ASD, CoA	y	y	y	y	y	y	n	n	n	y	y	Duplex kidney	*PTPN11*	Features of NS, delivery at 30 wk, female, 1750 g, death at day 21
2	18,28		n	y	TOF	n	n	n	n	y	y	n	n	n	y	n	Brachycephaly Cryptorchidism	*PTPN11*	Features of NS, isolated thrombocytopenia, term delivery, male, 2770 g, death at 11 months
3	12,17, 20	y	y	y	VSD	n	n	n	n	n	n	n	y	n	n	n		*BRAF*	Term, cesarean delivery, male, 3580 g, death at 10 months
4	13,17, 24	y	y	y	HCM, Right- sided AoA	y	y	y	y	n	y	n	y	n	n	n	Portosystemicshunt Posteriorly rotated ear	*RIT1*	Features of NS, delivery at 35 wk, female, 3130 g, death at 1 month
5	13,17, 21	y	y	y	VSD, Left SVC, HCM	n	n	n	n	y	n	n	n	n	n	n		*LZTR1*	Features of NS, term delivery at 40 wk, 3215 g, survive
6	32		y	y		y	y	y	n	n	y	n	y	y	y	n	Hepatomegaly, Posteriorly rotated ear	*KRAS*	Thrombocytopenia, preterm delivery, female, death at day 20
7	10, 20, 23		y	n	VSD, PS, DORV	n	n	n	n	n	n	n	n	n	n	n	Duodenal atresia	Typical NS	Features of NS, preterm delivery 33 wk, death at day 2.5
8	18, 19, 24		n	n	HCM	n	n	n	n	y	y	n	n	n	y	n	Abnormal head shape	Typical NS	Preterm delivery at 32 wk, 1450 g, web neck, cubitus valgus, wide shield chest, low hair line
9	13,18, 32	y	n	y		y	y	y	y	n	y	n	y	n	n	n		*PTPN11*	Preterm delivery, male, low-set ears, down slant pf, PPHN, bilat chylothorax
10	11,16, 26	y	y	y	HCM	n	n	n	n	n	y	n	n	n	n	y		Typical NS	Preterm delivery at 32 wk, male 2328 g, death at day 11
11	14,27, 31	n	y	y	HCM	y	y	y	y	y	y	n	n	n	n	n		Typical NS	Features of NS, preterm delivery at 33 wk, male, 2260 g, death at day 7
12	12,18	n	n	n	VH, PS	n	n	n	n	y	n	y	n	y	n	n		*PTPN11*	Features of NS, cesarean delivery at 38 wk, female, 3400 g, death at 2 months
13	13,21, 25	y	y	y	CoA	y	y	n	n	y	y	n	n	n	y	y	Ventriculomegaly, hypertelorism	Typical NS	Features of NS, cesarean delivery, male, 3100 g, survive

AoA: aortic arch; ASD: atrial septal defect; CH: cystic hygroma; CoA: coarctation of the aorta; DORV: double-outlet right ventricle; DV: ductus venosus; GA: gestational age (week); HCM: hypertrophic cardiomyopathy; LGA: large-for-gestational age; LV: left ventricle; PS: pulmonary stenosis; SUA: single umbilical artery; SVC: superior vena cava; TNF: thickened nuchal fold; TNT: thickened nuchal translucency; TOF: tetralogy of Fallot; VH: ventricular hypertrophy; VSD: interventricular septal defect; Typical NS: typical neonatal features of Noonan syndrome based on review by geneticists. * Nos. 1–10: cases from Ramathibodi Hospital; Nos. 11–13: cases from Maharaj Nakorn Chiang Mai Hospital.

### 3.2. Literature Review (Summarized and Presented in Table 2)

A total of 31 reports and our series were included, consisting of 105 fetuses of Noonan syndrome meeting eligible criteria and available for analysis, as presented in Supplementary Table S1 [12,14,15,17,19,20,21,23,24,25,26,27,28,29,30,31,32,33,34,35,36,37,38,39,40,41,42,43,44,45,46]. Of them, 50 fetuses underwent first trimester ultrasound, and 94 underwent second/third trimester ultrasound. Note that some fetuses underwent both first trimester and second/third trimester ultrasound.

Some similar abnormal findings were presented differently in various reports and were grouped as one entity, such as interventricular septal hypertrophy, left ventricular hypertrophy, and HCM. Hydrops fetalis without specifying the location of fluid collection was reported in many studies and was presented in this review as fluid collection in at least on body space, in addition to hydrops fetalis, while pleural/pericardial effusion, ascites, and subcutaneous edema are presented as missing values. Dilated jugular lymph sac was considered as cystic hygroma.

The results may be summarized as follows:

(1). Lymphatic drainage disorders (in both the first trimester and second/third trimesters)
  The most consistent finding in the first trimester was thickened nuchal translucency, accounting for 71.4% of cases, followed by cystic hygroma (16.3%). The most common consistent findings in the second/third trimesters was fluid collection in at least one body space, accounting for 59.3% of cases (including hydrops fetalis; 46.2%), followed by cystic hygroma (33.0%), and thickened nuchal fold (26.4%).
(2). Cardiac abnormalities (in the second/third trimesters)
  Relatively specific abnormalities included ventricular hypertrophy (33.7%) and pulmonary stenosis (13%), and other non-specific disorders included VSD (12%) and small left heart side (9.8%). Note that ventricular hypertrophy exhibited a wide range of severities, ranging from localized to diffused hypertrophy and eventually HCM.
(3). Polyhydramnios (48.4%)
(4). Other associated-anomalies with low frequencies

Notably, pyelectasis was also a common prenatal feature, accounting for approximately 20% of cases, while the distinctive craniofacial features commonly found postnatal life were identified in only a small portion of cases. Interestingly, some rare sonographic features found in general practice were relatively common in this review. However, uncommon findings, including absent ductus venosus, short femur, and single umbilical artery, were also noted.

**Table 2:** Frequencies of prenatal sonographic features among 105 cases of Noonan syndrome.

	Number (n)	%
**First trimester (N:49)**		
Thickened nuchal translucency	35	71.4
Cystic hygroma	8	16.3
**Second/third trimesters (N:91)**		
Cystic hygroma	30	32.6
Thickened nuchal fold	24	26.1
Cardiac abnormality		
■ *Ventricular hypertrophy*	30	33.0
○ *Ventricular/interventricular septum hypertrophy*	*23*	*25.3*
○ *Hypertrophic cardiomyopathy (poor contractility)*	*7*	*7.7*
■ *Pulmonary/pulmonic valve stenosis*	12	13.2
■ *Interventricular septal defect (VSD)*	11	12.1
■ *Small left side (aortic stenosis, hypoplastic left heart, coarctation of the aorta)*	9	9.9
■ *Valve dysplasia*	3	3.3
■ *Others*	13	14.3
Fluid collection > 1 space	54	59.3
■ *Hydrops (> 2 spaces)*	42	46.2
■ *Pleural effusion*	21	23.1
■ *Subcutaneous edema*	10	11.0
■ *Ascites*	6	6.6
■ *Pericardial effusion*	8	8.8
Polyhydramnios (N: 92)	46	50.0
Pyelectasis (N: 91)	18	19.8
Short femur (N: 92)	9	9.8
Large-for-gestational age (91)	10	10.9
Low-set ears (N: 91)	7	7.7
Frontal bossing (N: 91)	7	7.7
Absent ductus venosus (N: 91)	6	6.6
Single umbilical artery (N:91)	6	6.6
Hypertelorism (N: 91)	6	6.6

## 4. Discussion

In summary of the gained insights from this review, the consistent findings among various reports are as follows: (1) Lymphatic drainage disorders: The most consistent finding is thickened NT in the first trimester, followed by various features of fluid collection in body spaces in the second/third trimesters (either cystic hygroma, pleural/pericardial effusion, ascites or skin edema), with a wide spectrum of severity, ranging from simple increased NT in early gestation to hydrops fetalis in late gestation. (2) Cardiac abnormalities: The most consistent finding and relatively specific findings were ventricular hypertrophy, ranging from focal hypertrophy, such as interventricular septum or left or right ventricular wall, to HCM and heart failure, as well as pulmonary stenosis. Other less specific abnormalities are also increased, especially VSD and small left-sided heart. (3) Polyhydramnios in late gestation were commonly noted. (4) Other associated anomalies with low frequencies include pyelectasis, short femur, absent ductus venosus, single umbilical artery, and distinctive craniofacial appearances.

The prenatal natural course of Noonan syndrome can be delineated, extending the phenotype of the disease to prenatal life. Several findings are prenatally distinct. The sonographic findings seem mainly caused by the consequences of lymphatic drainage obstructions and cardiac defects. Notably, signs of lymphatic drainage obstruction are more obvious and sensitive during fetal life, while signs of cardiac hypertrophy are more obvious during postnatal life.

Based on this review, lymphatic disorders are typical prenatal features of Noonan syndrome, expressed with varying degrees of severity, ranging from simple thickened NT or cystic hygroma in the first trimester to fluid collection in various body spaces and eventually hydrops fetalis. Accordingly, the detection of any manifestation of lymphatic drainage obstruction must call for detailed ultrasound examination, including fetal echocardiography and karyotyping. Of fetuses with lymphatic drainage disorders with normal karyotyping, Noonan syndrome must be listed in the differential diagnosis, and RASopathy genetic testing must be performed, especially in cases associated with abnormalities commonly seen in the syndrome mentioned above.

Some abnormalities might be more obvious with advanced gestational age or after birth. Note that the frequency of hypertrophic cardiomyopathy was diagnosed in only 8.7% of cases, while the frequency in postnatal life was reported to be approximately 25% [7]. In fact, postnatally, cardiac abnormalities of all types could be identified in as many as 60–90% [20,47]. The much lower detection rate of cardiac abnormalities in fetal Noonan syndrome is likely explained by the fact that the existing cardiac abnormalities were still subtle during early gestation, gradually became more obvious with advancing gestational age, and were most obvious in postnatal life. Certainly, mild and localized ventricular hypertrophy in early gestation could readily be missed, and it is likely diagnosed later in postnatal life. Therefore, fetuses with Noonan syndrome without prenatally detected cardiac anomalies should undergo postnatal serial echocardiography for earlier diagnosis in neonatal life.

Both pulmonary stenosis and HCM often develop late in gestation or even after birth. They typically evolve or have progressive changes from mild to severe forms in late gestation or after birth, making prenatal diagnosis challenging [48,49]. About half of those with HCM are detected by age 6 months, and the remainder are detected mostly before 5 years of age [6]. Therefore, when ventricular hypertrophy is suspected, objective measurements should be done to confirm, and follow-up assessments of progressive changes and cardiac function must be performed for early detection of cardiomyopathy.

Interestingly, many uncommon sonographic signs are likely associated with Noonan syndrome rather than coincidental findings. Specifically, pyelectasis was identified in nearly 20% of cases, compared to only 1.7% among more than 60,000 fetal scans [50]. Likewise, single umbilical artery was demonstrated in 6.5%, which is much more than the 1.3% noted in normal fetuses [51]. Importantly, absent ductus venosus was identified in 5.5%, while the prevalence in general population is approximately only 0.6% [52]. Thus, such sonographic features should be considered as supporting signs suggestive of Noonan syndrome; however, isolated findings may be less helpful. Additional supporting findings are also important, such as distinctive facial characteristics of hypertelorism, flattened nasal ridge, and low-set ears. These findings are not routinely evaluated and sometimes require high levels of expertise. In cases with a high index of suspicion, it is worthwhile to seek out all of these ultrasound clues.

**Clinical implication:** Prenatal diagnosis is important for counseling the couples and offering choices for management and decision-making. Since Noonan syndrome has a relatively specific pattern of prenatal phenotypes, we propose that the scoring system for the diagnosis of Noonan syndrome in neonates should include the common prenatal sonographic ultrasound finding, especially thickened NT in euploid fetuses, unexplained lymphatic drainage obstruction either expressed by non-immune hydrops fetalis or a single body space of fluid collection in the second/third trimesters, HCM, pulmonary stenosis, and polyhydramnios. Notably, several prenatal findings were more obvious than those note in postnatal life, especially fluid collection in body spaces. Moreover, some very useful prenatal findings could not be evaluated in postnatal life, such as thickened NT, polyhydramnios, absent ductus venosus, and single umbilical artery.

A comprehensive scoring system for the diagnosis of Noonan syndrome [53] should include the prenatal phenotype; this might be important for diagnosis, especially in the settings where molecular diagnosis is not available. Our suggestion is based on the fact that most patients with Noonan syndrome have some relatively specific prenatal phenotypes, facilitating the early diagnosis of Noonan syndrome, especially in severe cases, and proper management. Compared with postnatal phenotypes, lymphatic disorders are more obvious in prenatal phenotypes, and polyhydramnios is a feature detected only in utero. In contrast, cardiac abnormalities and distinctive facial appearance are more readily appreciated in postnatal life.

**Differential diagnoses:** Making a differential diagnosis based on prenatal ultrasound is challenging. According to our series and a review of the literature, fetuses with Noonan syndrome display a variety of sonographic features. Nevertheless, it tends to have a specific pattern of sonographic findings that can assist in making a diagnosis. Several syndromes are included in the differential diagnosis due to shared features, such as congenital heart disease, lymphatic abnormalities, short stature, and facial dysmorphism, which overlap with Noonan syndrome. Genetic testing is often necessary for a definitive diagnosis. The differential diagnosis for fetal Noonan syndrome may include a variety of genetic and syndromic disorders with overlapping prenatal features, including: (1) Turner syndrome: This is the most common differential diagnosis as they share common sonographic features such as thickened nuchal translucency, cystic hygroma, and some cardiac defects, especially coarctation of the aorta and pulmonary stenosis. However, Turner syndrome is typically not associated with cardiomyopathy. (2) Cardiofaciocutaneous (CFC) syndrome: Typical features include cardiac defects (pulmonary stenosis, hypertrophic cardiomyopathy), facial dysmorphism, skin abnormalities, polyhydramnios, and macrosomia. (3) Costello syndrome: Typical features include coarse facial features, fetal growth retardation, cardiac anomalies (such as hypertrophic cardiomyopathy), and an increased risk for tumors (rhabdomyosarcoma, neuroblastoma). (4) Skeletal dysplasias (e.g., achondroplasia): These conditions are characterized by shortened long bones, facial dysmorphism, and, in some cases, hydrocephalus. However, limb shortening, a key feature in skeletal dysplasias, is not typical in Noonan syndrome. (5) Fetal hydrops: Prenatal features include fluid accumulation in at least two body spaces, such as subcutaneous edema, ascites, or pleural effusion. The causes of hydrops are broad and can include fetal anemia, cardiac failure, infections, or other genetic syndromes. (6) Williams Syndrome: Main features include congenital heart defects (supravalvular aortic stenosis), facial dysmorphism, and growth restriction. (7) CHARGE Syndrome: Main features include coloboma, heart defects, choanal atresia, growth retardation, genital abnormalities, and ear anomalies.

**Limitations** of this study are as follows: (1) Second and third trimester findings could not be clearly defined since the authors did not specify the timing of the first appearance of these findings given that examinations were performed multiple times over different weeks. (2) Given the study’s extended time frame, the quality of the ultrasound machines must have affected the detection rates of some subtle abnormalities, such as localized hypertrophy of the ventricle. Note that ventricular hypertrophy was very rarely detected in cases reported before 2000. (3) Several sonographic signs in various reports might have been based on different criteria, such as the width of the renal pelvis to define pyelectasis, ventricular wall thickness for the diagnosis of hypertrophy, etc.

## 5. Conclusions

This review better characterizes the sonographic features of fetal Noonan syndrome based on a larger sample size, illustrating a wider spectrum of prenatal phenotypes, including lymphatic drainage disorders, cardiac abnormalities, polyhydramnios, and absent ductus venosus.

## Data Availability

The datasets analyzed during the current study are available from the corresponding author upon reasonable request.

## Supplementary Materials

The following supporting information can be downloaded at: https://www.mdpi.com/article/10.3390/jcm13195735/s1.
